# Medical Items and News of Interest to the Physician

**Published:** 1875-01

**Authors:** 


### For the Use of Physicians.

CULLED FROM THE STANDARD MEDICAL JOURNALS OF THE WORLD.

NOTE.—These formulæ are intended only for the regular practitioner of medicine, and should be employed only by him, or under his immediate supervision.

**A New Symptom of Bromism.**

Dr. Hayden records in the *Irish Hospital Gazette* green vision as having been caused by bromide of potassium in a case under his care.

**Suppositories of Chloral.**

Are recommended by Dr. Const. Paul in cancer of the uterus. They are made from cacao butter, 11 grammes; white wax, 7 grammes; and chloral hydrate, 6 grammes; to be divided into six suppositories.

**Treatment of Erysipelas.**

The *Medical Record* states that in the Brooklyn City Hospital the following has proved efficacious as a local application in erysipelas: Acetate of lead, carbonate of magnesia, camphor, each twenty grains, water one pint.

**A Good Anodyne.**

An English journal gives this formula: Alcohol, one quart; gum guiac., one ounce; gums myrrh and camphor, and cayenne pulverized, each half an ounce. Mix and shake occasionally for a week or ten days, and filter or let settle for use. Apply freely for surface pains, or it may be taken in teaspoon doses for internal pains.

**Treatment of Eczema Papulosum.**

M. Bazin uses the following in cases of this form of eczema accompanied by dyspepsia, etc.:—

℞. Sodii bicarb. ʒ i. ℈i;

Syrupi saponariæ, f ℥ vij. M.

Sig.—Tablespoonful morning and evening.

Externally the following:

℞. Boracis, gr. xxx;

Glycerinæ, ʒ iiss;

Aquæ destillat, ad f ℥ x.

This lotion is to be applied, rather warm, morning and evening, and the skin is afterwards to be powdered with starch. Occasionally starch-baths—two pounds of starch to the bath—are found to give relief.

**Ethereal Tincture of Phosphorus.**

Phosphorus, 1 part;
Ether, 50 parts.

Macerate for a month in a bottle covered with black paper; decant into small vials similarly protected. Dose, five to ten drops in some emollient liquid.—*Paris Codex.*

**Phagedena.**

In the Penn. Hospital they use Bristow's solution of bromine, and follow it by yeast and charcoal poultices. The solution is made as follows:

℞

Bromine, ʒ j;

Bromide of potassum, ℈ ij;

Aquæ ʒ ij. M.

**Pathognomonic Sign of Pertussis.**

The practitioner may be sometimes consulted on a case of hooping-cough without having the opportunity of witnessing a paroxysm. In such a case M. Bouchut recommends him to examine the frænum linguæ, which he will always find the seat of a small ulcer in children the subjects of pertussis, or who are on the point of becoming so.—*Revue Medico-Photographique.*

**Chronic Diarrhœa.**

Rayer (*Union Medicale,* No. 73), recommends the following combination in the treatment of this complaint:

℞

Bismuthi Subnitratis, ʒ i;

Cinchonæ flavæ, pulv. ʒ ss;

Carbonis Ligni, ʒ i.

Misce et in chartulas XX divide.

S. One powder two or three times daily, in the intervals between meals.

**Application for Burns.**

M. Lebigot, in the London *Lancet,* recommends the following mixture as having been very successful: Cape aloes, four ounces; water, ten ounces; alcohol (90°), three ounces. The ingredients are to be melted together in a china plate over a slow fire, allowed to cool, and then filtered; after which three more ounces of alcohol are to be added. It is then ready for use. A tablespoonful of the liquid mixed with a teaspoonful of acetate of lead and twenty tablespoonfuls of water constitutes an excellent remedy. It is to be applied morning and evening on the burnt parts.

**Burns.**

The surgeons of Roosevelt Hospital use the following prescription with gratifying results.

℞

Tannic acid, ℈j ;

Chloroform, gtt. xx ;

Simple cerate, ℥j. M.

Spread upon lint and cover the parts affected.

**Oxide of Zinc for Night Sweats.**

The *Pacific Medical and Surgical Journal* remarks that the most ancient and venerable remedy for night sweats is aromatic sulphuric acid, in infusion of cincrona, serpentaria, or sage. The best of all remedies, however, is this: Oxidi zinci, gr. xxx.; ext. hyoscyami, gr. xv. M. f. pil. x. Sig. Take one at bedtime.

**Chronic Cystitis.**

At the Penn. Hospital they inject a tablespoonful of the following mixture, in a pint of water, three times daily :

℞

Borax, ℥j ;
Glycerine, ℥vj. M.

They also give the patient to drink of an infusion of triticum repens and bucu.

**Sick Headache.**

Dr. R. H. McKay, of Tennessee, writes to the *Medical and Surgical Reporter:* For the benefit of those who suffer with headache, I ask you to publish the following formula, the efficacy of which I have tested time and again: Granulated muriate of ammonia, one teaspoonful ; morphiæ acet., gr. j.; water, ℔ ss. Sig. Dose for an adult, two teaspoonfuls every ten minutes (precisely) till relief is obtained.

**Tonic Prescription.**

The following is regarded specially beneficial as a general tonic in syphilitic patients, and has been found to act exceedingly well in those cases in which the functions of the stomach have been almost destoyed by the use of alcoholics:

℞

Quina sulph. gr. xxx ;

Tr. ferri chloride ʒiij ;

Strychnia sulph. gr. ¼ ;

Syrp. zingiberis et.

Aquæ ad. ℥iij.

Sig.—Teaspoonful every four hours.

**Chilblains and Coryza.**

The following is a very convenient, economical, and efficacious application for chilblains and chaps:—Alcohol at 85°, 100 parts; glycerine, 25 parts; and phenic acid, 1 part. Powdered camphor sprinkled with tincture iodine, and inhaled by the nostrils, constitutes one of the most prompt and certain of remedies in coryza, or "cold in the head."—*Revue Medico-Photographique.*

**Cure for the Toothache.**

Dr. Henry T. Reynolds, of Baltimore, writes to the editor of the *Medical News,* that for eighteen months he has been using acetate of lead as a remedy for toothache. He finds it better than any of the numerous remedies proposed in the books, and in cases in which it is applicable, the relief is instantaneous. He advises the sufferer to apply from one to three grains to the cavity for a moment or two, then spit it out. It fails in fewer cases than any remedy that Dr. Reynolds ever tried, not more than eight per cent.

**Pherchloride of Iron in the Treatment of Hæmorrhiods.**

A writer in the *Dublin Journal of Medical Science* states that, having under his care a severe case of "bleeding piles" where all former treatment, including applications of fuming nitric acid, had been of no avail, he concluded to try injections of perchloride of iron. For this purpose twenty minims of the ordinary tincture were injected into each mass by means of a hypodermic syringe. The injection caused less pain than the nitric acid, and one administration sufficed to remove the hæmorrhoids completely.

**Caution in the Use of Ergot.**

Dr. Wernich maintains that ergot considerably accelerates secretion of urine. He relates two cases in support of his experiments, in which the rapid formation of urine, under the influence of the drug, was such as to render parturition impossible. After catheterism had been effected, labor took place speedily. As a practical conclusion, Dr. Wernich lays down the rule that in all cases of labor in which ergot of rye has been administered, the condition of the bladder must be looked into before any manipulation is performed, and catheterism should always be performed in case of arrest of labor.

**Bronchitis.**

Dr. Tanner of Bolivar, Mo., writes the Phila. *Med. & Surg. Reporter*, recommending the following formulæ in the treatment of this affection :

℞

Iodide pot., ʒ ij.

Tr. ferri chloridi, ʒ j ;

Syrp orange peel, ℥ ij ;

Aquæ. f. ℥ iv. M.

Sig : A tablespoonful three times daily.

---

**Catarrh of the Bladder.**

His formula is as follows :

℞

Iod. pot. ℥ ss ;

Holland gin ℥ iv ;

Tr. cubebs, ℥ ij. M.

Sig :—Teaspoonful three times daily, for three or four days, then intermit three or four days, then resume again the same length of time, and so on until cured.—*Ibid.*

---

**Pruritus Vulvæ.**

*La France Medicale* gives the following recipes:

℞

Alum, ʒ j.

Barley Water, ℥ vj. M.

For a lotion to be used three or four times daily, M. Harding frequently employed the following:—

℞

Corrosive sublimate, gr. xv;
Distilled water, ℥ iij;
Alcohol, q. s. M.

A spoonful in a glass of tepid water, to be applied to the parts, avoiding rubbing. In the pruritus which so often accompanies pregnancy, Danyan employs the following :

℞

Oxide of Zinc, ℥ j ;
Borax, ʒ ss ;
Simple Cerate, ℥ ss ;
Almond oil, q. s.
Hydrochlorate of morphia, gr. iijss. M.

M. Bazine prescribes this liniment :

℞

Lime water,

Glycerine, āā ʒ j ;

Almond oil ℥ ij M ;

He also advises the following formula :

℞

Glyceriue, ℥ v ;

Laudanum, ʒ j ;

Essence of Roses, gtt. v. M.

**Pertussis.**

The Boston *Medical & Surgical Journal* contains a communication from Dr. Spooner of Hingham, highly recommending Dr. Caldwell's treatment of pertussis, by inhalation. The formula given is as follows:

℞

Fld. extract belladonnæ, ♏ v. to x.;

Potass. bromid., ℈ j;

Ammon. bromid., ℈ ij;

Aquæ, f. ℥ ij. M.

Inhale one tablespoonful in the ordinary steam atomizer. Quite a number of cases are cited as being cured by this process.

---

**Glycerine Jelly.**

The London *Chemist* and *Druggist* remarks: "Glycerine jelly is usually cold cream tinted with a little rose oil and with some glycerine incorporated while it is warm. A more distinctive preparation is produced as follows:—

Transparent soap, 1 oz. (by weight.)
Water, - 4 oz. "
Glycerine, - 24 oz. "

Dissolve the soap in the water by heat, adding an equal quantity of glycerine. When dissolved, and while still hot, add the remainder of the glycerine. When nearly cold, perfume according to choice, and pour into glass jars. This is a transparent jelly of a pale amber color."

---

**Hydatids**

A writer in the *Australian Med. & Surg. Review* recommends from experience the use of turpentine in hydatids of the liver or brain. The following is the prescription given :

℞

Quinia sulph., gr. ⅓ ;
Terebinth. venet., gr. ijss ;
Pulv. rad. glycyr. q. s. M.
Ft. pil. no. j.

One such is to be taken three times a day before meals. It is also useful in tapeworm. Another prescription is :

℞

Turpentine, ℥ j ;

Tinct. opii. ʒj ;

Tinct. bucu., ʒ iv ;

Treacle ℥ iij.

Strong decoction Kousso, s. q. to make an ℥ viij mixture. Of this take a tablespoonful at first every three, then every four hours, and afterwards three times daily.

**Treatment of Pneumonia and Bronchitis by Carbolic Acid.**

Mr. Henry Greenway states, in the *British Medical Journal*, that he has been treating uncomplicated pneumonia and bronchitis very successfully with carbolic acid. The following is his formula for an adult:—

Glycerini acidi carbolici, ʒ ij.

Extracti opii liquidi, ♏ xxx;

Aquæ camphoræ, ℥ vj.

M. S. A tablespoonful every four or six hours, in water.

---

**Eczema Capitis.**

Cure it if possible, notwithstanding the somewhat prevalent idea to the contrary. The method of procedure recommended is as follows:

First, apply a poultice every night until all the scabs are removed. The ulcerations, which are sometimes present after the scabs have been removed, are best cured by the application of a wash made of nitrate of silver grs. v. to the ℥ i. of water. The following is then used with good success:

℞. Aquæ Cologne, ℥ iv;

Glycerine, ℥ ij;

Carbolic acid crystals, ʒ i;

Borax, ʒ i. M.

Continue the application of this remedy for some time for the purpose of curing the eczema.

---

**Antidote of Rabies.**

Dr. Jitzki communicated, in January, 1874, to the Imperial Society of Wilna (Russia), a fact of some interest. A very savage dog was in the habit of killing vipers (*Coluber berus*), and his mouth and neck were covered with wounds inflicted by the vipers. This animal was bitten by a mad dog, which had already bitten several horned cattle and a dog; these all perished in a rabid state. The owner of the first-mentioned dog, not wishing to destroy him, determined, however, to watch him, and made up his mind to kill the poor animal at the first symptom of rabies. Nothing, however happened, and the dog remained quite well.

This case struck Dr. Jitzki, especially as he learned that a woman of the same locality had been bitten by a viper and some time afterwards by a mad dog without suffering from hydrophobia. He was thus led to suspect that there is antagonism between the virus of hydrophobia and the virus of vipers. If this were true, young dogs might be inoculated with the latter virus, and thus be made insusceptible to hydrophobia.—*The Lancet.*

---

**Brilliant Colors for Druggists' Show Bottles.**

The London *Chemist and Druggist* gives these formulæ for the purpose: *Royal Blue:* Take nitrate of copper 3 oz., water sufficient to dissolve it; add water of ammonia as long as the color becomes deeper, then water to make up two gallons. *Emerald Green:* Nickel, 3 oz.; muriatic acid, 4 oz.; nitrous acid, 2 oz.; digest for twenty-four hours, add 2 gallons of water, and filter. *Crimson:* Iodine and iodide of potassium, of each, 2 dr.; triturate with one dr. of water, then add 3 gallons of water and 4 oz. of muriatic acid. *Yellow:* Bichromate of potash, 2 dr.; hot water 4 oz. dissolve and add 4 oz. sulphuric acid and 2 gallons of water. An American writer states that he produces a red, white and blue, in one bottle, by putting in first, water colored blue, then tupentine left white, then alchohol colored red.

---

**Delirium Tremens.**

What this class of patients most need is food and rest. Generally, if rest can be secured, there will be no further trouble in the management of the patients, for they will return to taking food, and in a short time the immediate effects of their debauch have passed.

Rest is usually secured by the administration of either potass. bromid. in large doses, grs. xxx. every two hours perhaps, or chloral hydrat. grs. xx. every hour. It is necessary in using the latter remedy to be cautious with regard to the size of the first doses, until the strength of the preparation and the susceptibility of the patient is fully understood. Then it may, perhaps, be given in much larger doses than indicated. Occasionally even large doses will be of no avail.

A very common prescription employed is the following:

℞. Potass. bromid, ʒ iss;

Chloral hydrat, ʒ iss;

Syr. aurantii;

Aqua, āā ℥ ij.

M.—S. Half fluid ounce, and repeated every hour if necessary to procure sleep.

**Diarrhœa of Phthisis.**

In the Philadelphia Hospital they use the following formulæ :

℞

Opium, ½ to 1 gr.

Bismuth subnit., gr. xv;

Ipicac, ½ gr.

Taken pro re nati.

℞

Nit. Silver, ¼ to ½ gr.
Opium, ½ to 1 gr. M.

Ft. pil. to be taken p. r. n.

℞

Strychnia, sulph 1-24 gr.
Bismuth subnit, 15 to 20 gr.

Taken p. r. n.

---

**Pleurisy with Effusion.**

Diuretics are mainly employed, but particularly furuginous diuretics. The liq. ferri per acetatis or Basham's mixture is uniformly administered. Diet sustaining. The formula for Basham's mixture as furnished by the apothecary of the Hospital is as follows ;

℞

Liq. ammon. act., ℥ vj ;

Acid acetic, ʒ iij ;

Tr. ferri chloridi, ʒ v ;

Alcohol, ℥ ij ;

Syrupus, ℥ iv ;

Aquæ ℥ iv M.

Sig :—Teaspoonful doses three or four times daily, or oftener. Paracentesis is never resorted to early, unless there are present some special symptoms which demand it.—*Penn. Hospital Reports.*

---

**Syphilitic Lichen.**

Good food and porter ; vapor bath not lasting more than ten minutes, once or twice a day ; and the following drugs :

℞

Potassio tartrate of iron, ʒ vj ;

Aquæ ℥ viij. M.

Teaspoonful of this mixture with from ⅛ to 1-16 of a grain of bichloride of mercury, t. i. d. The mercury is combined, not for the purpose of producing its specific effect, but simply for its tonic and alterative influence. Salivation is not desirable. In connection with the above the patient is also given Fowler's solution, gtt. xv. t. i. d. administered in milk and after meals. *The arsenic is commenced in doses of five or six drops*, and increased until *some nausea* is produced.—*Penn. Hospital Reports.*

**Night Sweats of Phthisis.**

Atropia and oxide of zinc are the two remedies chiefly employed for the relief of this particular symptom. Consumptive patients in general are placed upon Niemeyer's pill. The formula for the pill is as follows :

℞

Pulv. herb. digitalis, ℈ ss ;

Pulv. rad. ipecac;

Pulv. opii puri. āā gr. v ;

Ext. helleni, q. s. w : f. pil. No. xx ;

Cons. pulv. iric. flor.

Sig. one pill t. i. d.

—*Penn. Hospital Reports.*

---

**Facial Erysipelas.**

Tr. ferri chloridi, gtt. xx every two hours from the beginning. The common topical application is mucilage of slippery elm, keeping the parts constantly wet with it.—*Penn. Hospital Reports.*

We have had excellent success in the treatment of phlegmonous erysipelas of the face from the administration of liq. perchloride of iron in from five to ten drop doses, every three hours, and the topical application of cranbery poultices. The cranberries were cooked until soft enough to be crushed through a cullender and then applied every hour, cold, to the bare skin. This application gives instant relief to the burning and itching, and stops the swelling and spreading of the disease.—*Ed. Bistoury.*

---

**Ergotine in Internal Hemorrhage.**

Dr. Townsend, in the *Dublin Medical Journal*, recommends the hypodermic use of ergotine in various internal hemorrhages. He gives various instances of its use, of which we give two.

Case I. This case was a patient in the South Infirmary, named Mannix, who was admitted from Passage with hæmoptysis. He had been in India. His appearance was that of a man of intemperate habits. There was moist crepitation under left clavicle, also evident dilatation of arch of aorta ; the impulse over the sternum was very strong; there was no other symptom to indicate aneurism except an occasional pain in his back. As the blood was bright red, and coming up rather freely, with bubbling in the throat, I injected four grains of ergotine over his right elbow. His bowels had been well acted on before I saw him. The injection caused slight redness and some smarting, but completely checked the expectoration of blood. He did not spit any red blood afterwards, and only a few rusty sputa. One injection was sufficient in this case.

Case. II. P. Connell, a night watchman, was admitted into hospital with cardiac disease of old standing, and an attack of acute dropsy, caused by exposure to wet. The urine was highly albuminous. He improved under treatment and was able to sit up, when he began to pass pure blood from the kidneys. I injected four grains of ergotine with very little effect, as the urine was just as bloody next day. I then injected five grains, and he immediately complained of his arm, which was soon red and painful around the part injected. This had the desired effect, as the urine was passed quite free from blood, clear and healthy-looking, but still containing albumen. This continued for nearly a week, when the red color again appeared ; another injection had the same effect, and I then put him on nitric acid and iron. He had no return of the hæmaturia. He left the hospital, and died shortly after of congestion of his lungs, caused by exposure to cold outside.

**Neuralgia.**

One of the best methods of treating neuralgia is that recommended by Trosseau. A tight-top thimble is filled with cotton wool, and a few drops of strong aqua ammonia dropped on it. The open mouth of the thimble is then applied over the seat of pain for a minute or two, until the skin is blistered. The skin is then rubbed off, and upon the denuded surface a small quantity of morphia (gr. ¼) is applied. This affords almost instant relief. A second application of the morphia if required, is to be preceded by first rubbing off the new formation that has sprung up over the former blistered surface.

Dr. J. Knox Hodge recommends the following as an application which will relieve facial or any other neuralgia almost instantaneously:

℞

Albumen of egg, ʒ j;
Rhigolene, ℥ iv;
Oil of Peppermint, ℥ ij;
Collodion; chloroform, āā ℥ j. M.

Agitate occasionally for twenty-four hours, and by gelatinization a beautiful semi-solidified opodeldoc-looking compound results, which will retain its consistency and hold the ingredients intimately blended for months. Apply by smart friction with the hand, or gently with a soft brush or mop along the course of the nerve involved.

**Phosphate of Lime and the Juice of Raw Meat in the Treatment of Phthisic.**

Dr. H. Blanc, in the *London Lancet*, from extended experience, states that the use of the juice of raw beef with the phosphate of lime, is of great value when given in a form suitable to the patient and the disease. The meat juice is prepared thus: A pound of fresh beef deprived of fat, bones, etc., is placed over a quick fire for a few moments, in order to whiten and harden the external surface only. The meat is then cut into two or three pieces corresponding to the size of the meat press, and all the juice extracted by the pressure of a powerful screw. A pound and a half of meat gives a teacupful of juice. This should be prepared fresh daily. This juice, having all the physical properties of raw meat, is easily digested, is well tolerated, and, served in the following manner, is always very grateful to the patient : The juice should be mixed with equal parts of tepid broth made of bones and flavored with salt and pepper, to which tapioca, etc., can be added. Care, however, should be taken that the broth is never more than *tepid*, otherwise coagulation takes place, and the desired effect is not obtained.

The following is the method of treating a consumptive patient: Early morning, warm milk (not boiled), with bread and butter, and if the appetite be good, some fat bacon and eggs. At eleven or twelve o'clock, breakfast, before which a drachm of the syrup of triple phosphate should be taken. During the meal itself a dose of muriatic phosphate of lime, and half of the daily allowance of the raw meat juice in some broth. The meal should consist, according to appetite and digestive powers, of fish or poultry, or white meats, fresh vegetables, and a few glasses of wine. Dinner at six, on the same principles, broth with the remainder of the raw meat juice, and a desertspoonful of cod-liver oil. The muriatic solution should also be taken at dinner. At night, before retiring to rest, a cupful of warm, fresh milk. All hygienic rules, out-door exercise, ablution of skin, etc., should be carefully observed. Under this treatment the appetite rapidly returns, the cough becomes less troublesome, the expectoration lessens, the night-sweats and all unfavorable symptoms decline and disappear, the patient gains flesh and strength, and once more enjoys life.

**Hay Fever.**

G. W. A. (Tenn.) writes: The new remedy recommended by Dr. Dobell, which appeared in the August number of the *Pharmacal Gazette*, for the above named complaint, suggests the propriety of sending the following formula, that is not entirely new here, and has proven itself successful in every instance:

℞
Sol. quinine, 1 gr;
Aqua, 740 grs.

This is to be applied with a short, bushy, camel-hair pencil, at pleasure, in a similar manner as described by Dr. Dobell, or, by still better means, the nasal douche.

This preparation has the advantage over Dr. Dobell's in being more cleanly, and devoid of the nauseous castor-oil. The minute portion of the active ingredient may savor a little of homœopathy, but nevertheless it has shown itself an excellent remedy against the distressing complaints of the hay-making season. Since writing the above, our attention has been called to an article in a late number of the *London Medical Record*, by Prof. Binz, of Bonn, giving Dr. Helmholtz's history of his own case of hay fever, and the subsequent discovery of the use of quinia sulph. in the treatment of hay fever, or *Catarrhus Æstivus*. In a letter to Prof. Binz, Dr. Helmholtz says: "The curious dependence of the disease on the season of the year suggested to me the thought that organisms might be the origin of the mischief. In examining the secretions, I regularly found certain vibrio-like bodies in it, which at other times I could not observe in my nasal secretions. When I first saw accounts of the poisonous action of sul. quinia upon infusoria, I determined at once to try the experiment with that substance. The desired effect was obtained immediately, and remained for several hours. I could expose myself to the rays of the sun without fits of sneezing, and the other disagreeable symptoms coming on. My first experiments with quinine date from the summer of 1867; this year (1868) I began at once, as soon as the first traces of the illness appeared, and I have thus been able to stop its development completely." The high authority whence this remedy comes recommends it to the sufferers of hay fever and the careful attention of medical men. —*Pharmacal Gazette.*

**Hydrate of Chloral for Venous Injection as an Anæsthetic.**

Dr. Oré, of Bordeaux, has addressed a communication to the Société de la Chirurgie on the injection of chloral into the venous circulation. He has gone so far as from two to six grammes. He believes there is no other anæsthetic so potent. The insensibility in the animals thus operated upon was so great that the electric current alone interrupted it. In a case of tetanus occasioned by the contusion of the left middle finger, he injected twice, at an interval of some minutes, nine grammes of chloral dissolved in ten grammes of water. At the second injection the sufferer fell into a deep slumber, the breathing became calm and regular, the pulse was 70, and the insensibility so complete that the nail of the bruised finger could be avulsed without a trace of suffering. Next day the injection was repeated, and by the third day the severity of the malady was much abated. M. Verneuil witnessed five recoveries in tetanus by the employment of venous injections of chloral, and M. Boinet two. Should this remedy be equally successful in other cases it would prove an infinitely desirable innovation in regard to the treatment of tetanus, commonly so intractable.

**Chloral and Bromide of Potassium in Chest-Affections.**

Dr. S. L. Abbott, of Boston, in a communication to the *Philadelphia Medical Times*, says: "With regard to the use of chloral and bromide of potassium together as a sedative in cases of chest-affection, I would say that I have repeatedly used this combination, and I dare say it has occurred to many others to employ it. During the present autumn, some of my patients who are victims of 'autumnal catarrh' have found great comfort from it. The addition of a little morphia adds to its efficacy, and in one instance at least, that of a lady who is very susceptible to the disagreeable after effects which opiates so often produce, the chloral bromide seem to prevent their occurrance entirely."

